# Tobacco BY-2 Media Component Optimization for a Cost-Efficient Recombinant Protein Production

**DOI:** 10.3389/fpls.2018.00045

**Published:** 2018-01-26

**Authors:** Suvi T. Häkkinen, Lauri Reuter, Ninni Nuorti, Jussi J. Joensuu, Heiko Rischer, Anneli Ritala

**Affiliations:** VTT Technical Research Centre of Finland Ltd., Espoo, Finland

**Keywords:** tobacco, BY-2, recombinant protein, cost evaluation, GFP-hydrophobin I

## Abstract

Plant cells constitute an attractive platform for production of recombinant proteins as more and more animal-free products and processes are desired. One of the challenges in using plant cells as production hosts has been the costs deriving from expensive culture medium components. In this work, the aim was to optimize the levels of most expensive components in the nutrient medium without compromising the accumulation of biomass and recombinant protein yields. Wild-type BY-2 culture and transgenic tobacco BY-2 expressing green fluorescent protein–Hydrophobin I (GFP-HFBI) fusion protein were used to determine the most inexpensive medium composition. One particularly high-accumulating BY-2 clone, named ‘Hulk,’ produced 1.1 ± 0.2 g/l GFP-HFBI in suspension and kept its high performance during prolonged subculturing. In addition, both cultures were successfully cryopreserved enabling truly industrial application of this plant cell host. With the optimized culture medium, 43–55% cost reduction with regard to biomass and up to 69% reduction with regard to recombinant protein production was achieved.

## Introduction

Use of plant cell cultures offers an attractive alternative for the production of biomolecules. Compared to field-grown plants, cell cultures can be cultivated in a controlled and contained environment. Plant cell culture technology is a fascinating tool for various biotechnological applications offering possibilities for optimizing production, independently of climatic or environmental effects. Plant cells display several unique features differing from microbial and mammalian cells, e.g., in glycosylation pattern (Strasser, 2016) and the diversity of small molecules produced (Verpoorte and Alfermann, 2013). However, often encountered hindrance in using plant cells in biotechnological platforms is the economic burden, i.e., the contained cultivation of plant cells is expensive mainly due to the specialized medium requirements and low multiplication rate of the cells. Tobacco BY-2 (*Nicotiana tabacum* cv. ‘Bright Yellow’) cell line is highly synchronizable and thus desirable for investigation of various aspects of plant cell biology and metabolism (Shaul et al., 1996; Goossens et al., 2003; Nagata et al., 2004). BY-2 cells divide rapidly and may increase their biomass up to 100-fold in 1 week. This is a clear advantage when it comes to large-scale applications. Rapid accumulation of biomass and easy transformation has made the BY-2 cells the most frequently used plant cell line for protein production (Doran, 2013). Proteins ranging from antibodies, enzymes, and therapeutic to technical proteins (Sakamoto et al., 2008; Kirchhoff et al., 2012; Reuter et al., 2017) have been successfully expressed in BY-2 cells. However, when it comes to industrial production, two factors must be specifically ensured: stability of the production host and the cost efficiency of the production process (Kurppa et al., 2017).

Plant cell cultures are notorious for exhibiting somaclonal variation, i.e., accumulating unwanted characteristics over prolonged maintenance periods leading to loss of productivity or metabolic competence (Larkin and Scowcroft, 1981). Cryopreservation is a method where cells or tissues are stored at ultra-low temperatures, usually in liquid nitrogen (-196°C), halting the whole metabolism and thus allowing cells to retain their properties unchanged (Engelmann, 2004). Even though successful cryopreservation methods for a number of plant species have been established, empirical experimentation is needed to optimize all steps in the cryopreservation procedure according to species-specific requirements and product targets.

The culture medium for tobacco BY-2 cells was described by Nagata and Kumagai (1999). The medium is based on the Murashige and Skoog’s (1962) nutrients, but it is supplemented with phosphate by threefold compared to common MS medium (Nagata et al., 1992). When it comes to recombinant proteins, nitrate metabolism has recently been in focus since it is an essential building block for amino acids. Indeed, supplementing the media with additional nitrogen has increased the yield of secreted antibody up to 150-fold, at the expense of the growth rate (Holland et al., 2010). Later Vasilev et al. (2013) used factorial design to optimize media for a cell line expressing a secretory antibody resulting in significant increase in yield and substantially reduced packed cell volume. However, until now the optimization studies have solely focused on improving response parameters, such as biomass and/or product yields, without evaluating the cultivation costs caused by expensive nutrients in the culture medium.

The aim of this work was to investigate protein production following cryopreservation, to identify most cost-relevant nutrients in the culture medium and to determine culture medium compositions resulting in cheaper production of either biomass and/or recombinant protein.

## Materials and Methods

### Cell Lines

*Nicotiana tabacum* BY-2 cell suspension culture established by Prof. Nagata at the University of Tokyo was maintained as described (Nagata and Kumagai, 1999). The generation of transgenic BY-2 lines expressing green fluorescent protein–Hydrophobin I (GFP-HFBI) fusion protein was described in detail in Reuter et al. (2014). The expression construct (Joensuu et al., 2010) included CaMV35S promoter and signal sequence to target GFP-HFBI expression to secretion pathway. KDEL retrieval sequence was used to sequester GFP-HFBI in endoplasmic reticulum. The BY-2 GFP-HFBI lines were maintained as calli on modified MS-medium (Nagata and Kumagai, 1999) supplemented with 25 mg/l kanamycin and were subcultured every 3–4 weeks by visually selecting only the most fluorescent fractions under UV-light to fresh plates. This subcloning was performed over a period of 3 years resulting in a very high expressing cell clone renamed as ‘Hulk.’ Suspension cultures were maintained in 50 ml of the modified MS-medium supplemented with 50 mg/l kanamycin and subcultured weekly by transferring 5% (v/v) of the culture to fresh media.

### Biomass Harvesting and Expression Analysis

Amount of GFP-HFBI and total soluble protein were measured from the freeze-dried samples (Reuter et al., 2014). Biomass was harvested by centrifugation and freeze-dried (0.2 mbar, 48 h; Christ Alpha 1-4 LD Plus, Osterode am Harz, Germany). Cells were disrupted using steel beads and a Retsch mill (MM301, Haan, Germany) and cell powder was mixed with extraction buffer (1× phosphate-buffered saline; 12 mM Na_2_HPO_4_ × 2H_2_O, 3 mM NaH_2_PO_4_ × H_2_O, 150 mM NaCl), 1 mM EDTA, 100 mM sodium ascorbate, and 0.4 μM leupeptine hemisulfate (Sigma–Aldrich, St. Louis, MO, United States), and insoluble material was removed by centrifugation. Concentration of TSP was measured using the Bradford assay (Bradford, 1976) with Bio-Rad reagent (Bio-Rad, Hercules, CA, United States) and bovine serum albumin (BSA; Sigma–Aldrich) as standard. Extracted proteins were separated by SDS–PAGE on Bio-Rad Criterion-TGX and Mini-PROTEAN precast gels. The GFP concentration in TSP was determined by fluorometry. The fluorescence of the diluted samples was determined at 485/527 nm using a VICTOR^2^ plate reader (Perkin Elmer, Waltham, MA, United States) at 12 nm bandwidth and 100 ms measurement time. Sample dilutions were compared to a standard curve constructed with purified GFP (BioVision, Milpitas, CA, United States).

### Cryopreservation of BY-2

Tobacco BY-2 and BY-2 ‘Hulk’ were cryopreserved with the modified method described by Ogawa et al. (2012). Briefly, before cryopreservation, the culture was pre-cultivated for 7 days. Packed cell volume (PVC) was adjusted to 30% by centrifuging (150 *g*, 3 min) and adding LSP solution (2 M glycerol, 0.4 M sucrose, 86.9 mM proline). The obtained suspension was incubated in standard growth conditions for 1 h, after which the cell suspension was divided in 1.8 ml cryo-vials in 1 ml aliquots. The samples were frozen with a controllable freezer (Planer Kryo 560-16, PLD Finland). The freezing profile was adjusted as follows: -5°C/min into 0°C, hold 5 min in 0°C, -0.5°C/min into -35°C, and hold 30 min in -35°C. After freezing, the samples were dipped into liquid nitrogen. The samples were stored in liquid nitrogen for 24 h, after which they were placed in a container filled with sterile water in a +40°C water bath. The vials were thawn for 2 min so that all visible ice was melted. Excess LSP solution was pipetted off and the cell slurry was placed on solid growth medium with two filter papers. The plates were sealed with parafilm and incubated in dark for 24 h. The cells, together with the filter paper, were placed on a fresh plate and the incubation was continued as above.

### Stability Evaluation after Cryopreservation

Cell suspension cultures of the cryopreserved cells and treated cells (i.e., cells treated with cryopreservation solutions but not frozen) were made suspending the cells from the plate (approximately 1 g FW) in 20 ml liquid growth medium. After 1 week, the growth curve experiment was started with 40 g (FW) suspended in 1-L culture medium. Suspension was divided in 50 ml aliquots in 250 ml Erlenmeyer flasks. Samples were taken at time points 3, 4, 5, 6, and 7 days in three replicates. Cells were collected by filtering and both cell and medium samples were separately frozen and freeze-dried. The experiment was repeated for the original cell suspensions (cryopreserved and treated cells) with 7-day-old cultures after weekly subculturing in the laboratory for 3 months.

### Culture Medium Optimization

A cost estimation was performed to identify the most expensive nutrients in the culture medium. This involved the evaluation of costs of macro- and microsalts, vitamins, and plant hormones (**Supplementary Table S1**). Price evaluation of the culture medium was performed based on the list prices of the nutrients provided by Sigma–Aldrich. No other factors such as production costs were included in the evaluation. Evaluation resulted in altogether seven compounds, which were selected as studied factors based on their final cost and relevance pointed out earlier by Vasilev et al. (2013): KNO_3_, NH_4_NO_3_, MgSO_4_^∗^2H_2_O, KH_2_PO_4_, CaCl_2_^∗^2H_2_O, Myo-inositol, and 2,4-D. The screening of the factors was performed with BY-2 clone ‘Hulk’ against biomass production and GFP-HFBI accumulation. The screening was carried out according to experimental design (DoE) created with Modde11 software (Umetrics, Sweden), the range of studied factors was -3…+1.3-fold compared to the original medium (**Table 1**). Thus, altogether 19 experiments were included in the DoE using fractional factorial design at two levels. The most promising medium composition in respect of biomass production was subjected to more detailed examination, i.e., the cost of the selected media was compared and for the most cost-efficient media, components were reduced from 100 to 50% levels in 10% intervals. These experiments were performed with both BY-2 cell line and ‘Hulk.’ In addition, a test-set was performed in which 2,4-D and myo-inositol were kept at the same level as in original medium (0.2 and 100 mg/l, respectively). Finally, the costs for the biomass and GFP-HFBI yields in the most cost-efficient media were compared to the original culture medium.

**Table 1:** Experimental design setup with the levels of salts (mg/l).

Experiment name	KNO_3_	NH_4_NO_3_	MgSO_4_⋅ 7H_2_O	CaCl_2_⋅ 2H_2_O	KH_2_PO_4_	Myo-inositol	2,4-D	Medium
N1	633	550	123	147	123	33	0.1	A1
N2	2470	550	123	147	481	33	0.26	A2
N3	633	2145	123	147	481	130	0.1	A3
N4	2470	2145	123	147	123	130	0.26	A4
N5	633	550	481	147	481	130	0.26	A5
N6	2470	550	481	147	123	130	0.1	A6
N7	633	2145	481	147	123	33	0.26	A7
N8	2470	2145	481	147	481	33	0.1	A8
N9	633	550	123	572	123	130	0.26	A9
N10	2470	550	123	572	481	130	0.1	A10
N11	633	2145	123	572	481	33	0.26	A11
N12	2470	2145	123	572	123	33	0.1	A12
N13	633	550	481	572	481	33	0.1	A13
N14	2470	550	481	572	123	33	0.26	A14
N15	633	2145	481	572	123	130	0.1	A15
N16	2470	2145	481	572	481	130	0.26	A16
N17	1551.5	1347.5	302	359.5	302	81.5	0.18	A17
N18	1551.5	1347.5	302	359.5	302	81.5	0.18	A17
N19	1551.5	1347.5	302	359.5	302	81.5	0.18	A17
N20	1900	1650	370	440	370	100	0.2	Original


### Statistical Analysis

Statistical analyses were performed with SPSS Statistics 22 (IBM) software, using one-way ANOVA and Tukey’s HSD unless otherwise indicated. In case the ANOVA criteria for homogeneity of variances were not fulfilled (Levene’s test *p* < 0.05), Tamhane’s T2 test was used.

## Results

### GFP-HFBI Production by ‘Hulk’

**FIGURE 1:**
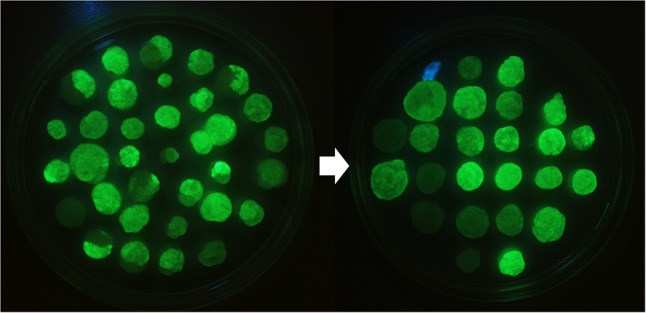
BY-2 lines expressing GFP-HFBI as a visually selectable marker. Only the most fluorescent cell fragments from the heterogeneous calli were subcultured and continuous selection over period of 3 years resulted in homogeneous BY-2 GFP-HFBI clones [Reprinted with permission of VTT Ltd., from Reuter (2016)].

**FIGURE 2:**
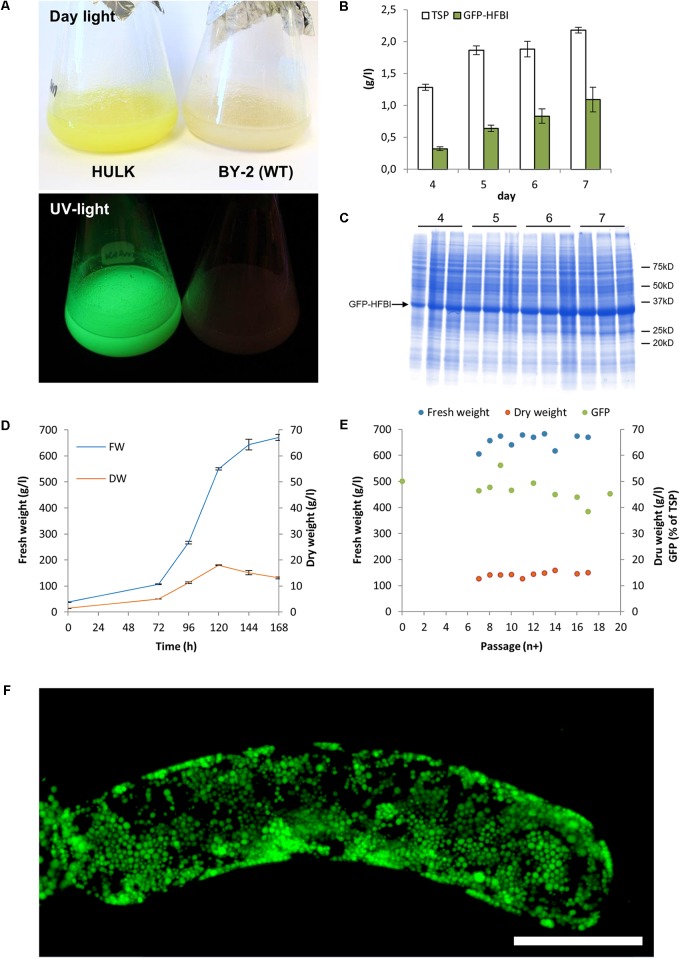
BY-2 cell line ‘Hulk’ expressing high levels of GFP-HFBI. **(A)** The accumulating GFP-HFBI is visible in the suspension cells with naked eye in daylight. **(B)** Protein accumulation over 1 week of cultivation. Error bars represent SDs (*n* = 3). **(C)** A Coomassie stained SDS–PAGE shows that GFP-HFBI (approximately 35 kD) builds the bulk of total protein. The parallel lanes represent three replicates. Same amount DW is loaded on the gel. **(D)** Growth of the cell line is similar to WT BY-2 cells. **(E)** Accumulation of fresh biomass, dry weight, and GFP-HFBI over follow-up period of 19 passages. **(F)** A z-stack of confocal microscope images showing accumulation of GFP-HFBI in protein bodies. Scale bar indicates 50 μm [Reprinted with permission of VTT Ltd., from Reuter (2016)].

The subcloning by passing only the most fluorescent cells further over a period of 3 years resulted in very homogenous BY-2 clones expressing GFP-HFBI (**Figure 1**). The best clone, named ‘Hulk,’ produced 1.1 ± 0.2 g/l (mean ± SD, *n* = 3) GFP-HFBI in suspension culture corresponding to 50.1 ± 8.5% of TSP or 8.3 ± 1.6% of the dry weight (**Figures 2A–E**). GFP-HFBI accumulated in condensed protein bodies (**Figure 2F**) as earlier observed (Reuter et al., 2014).

Accumulation of GFP-HFBI was followed over a period of 19 passages in suspension culture. This corresponded to a culture period of 5 months. Over this culture period, the yield remained between 44 and 56% GFP-HFBI of TSP. Notably during suspension culture period, the visual selection was not applied and this indicated that ‘Hulk’ had indeed stabilized and kept its high performance in accumulating GFP-HFBI.

### Cryopreservation of ‘Hulk’

**FIGURE 3:**
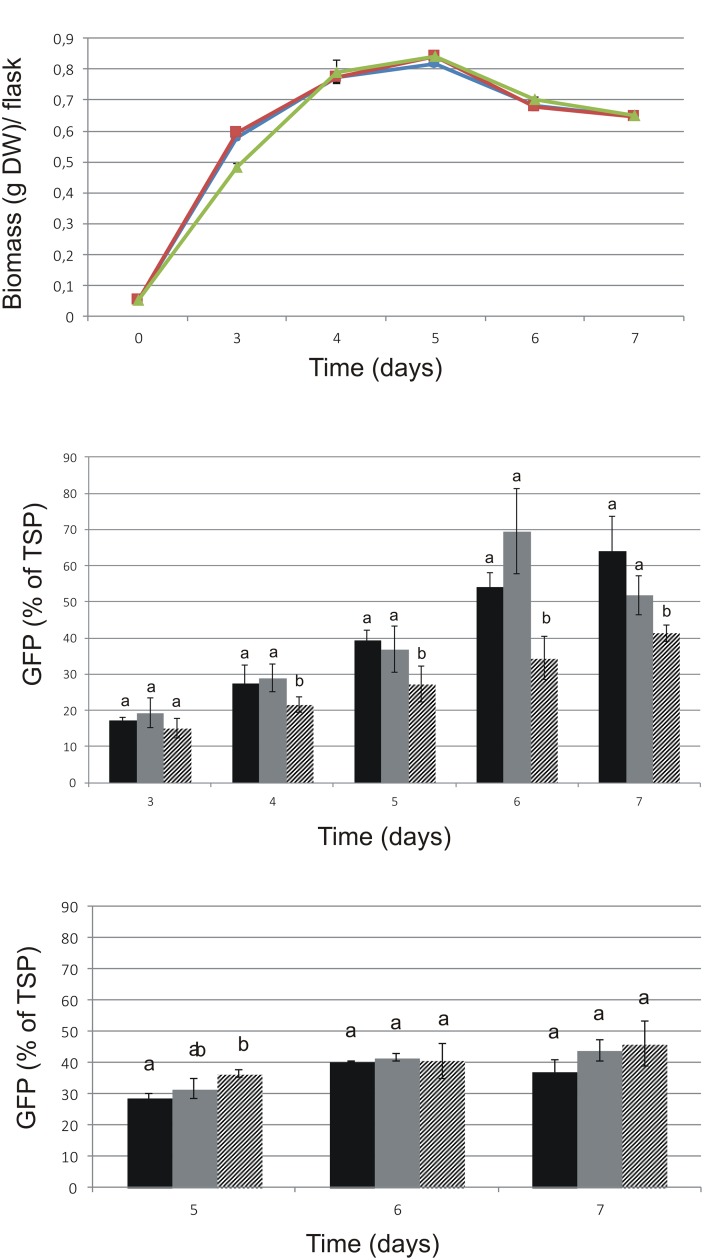
Growth and GFP expression of ‘Hulk’ after cryopreservation. Cultivation volume was 50 ml. Mean ( ± SD) of three biological replicates is shown. Letters indicate statistical differences of the three lines in each time-point with *p* < 0.01. **(A)** Comparison of the growth of ‘Hulk.’ Circle: after cryopreservation; square: treated with cryopreservation agents but not cryopreserved; triangle: original line. **(B)** GFP-HFBI measured in different time points before and after cryopreservation. **(C)** GFP-HFBI/TSP measured after 3-month maintenance. Black: cryopreserved; gray: treated, not cryopreserved; patterned: original culture.

The cryopreservation of ‘Hulk’ was successful and did not influence the growth rate after thawing (**Figure 3A**). GFP-HFBI expression was assayed before and after cryopreservation (**Figure 3B**). The levels of GFP-HFBI/TSP were higher in treated and cryopreserved samples in comparison with those of control samples from day 4 on. TSP levels in these samples were on similar range, without statistically different changes.

All cell lines were maintained as suspension cultures by regular subculturing for 3 months and the measurements were repeated in order to see whether the GFP-HFBI levels would remain stable during time. Notably, the visual selection was not applied during the subculturing regime. The differences seen right after cryopreservation (**Figure 3B**) were not visible after longer maintenance (**Figure 3C**), suggesting that cryopreservation and cryopreservation pre-treatment caused temporary stress to cells resulting in transient increase of GFP-HFBI expression levels. The differences between the treated, cryopreserved, and control lines were not significant after 3 months regular subculturing.

### Design of Experiments (DoE) – Culture Medium

**FIGURE 4:**
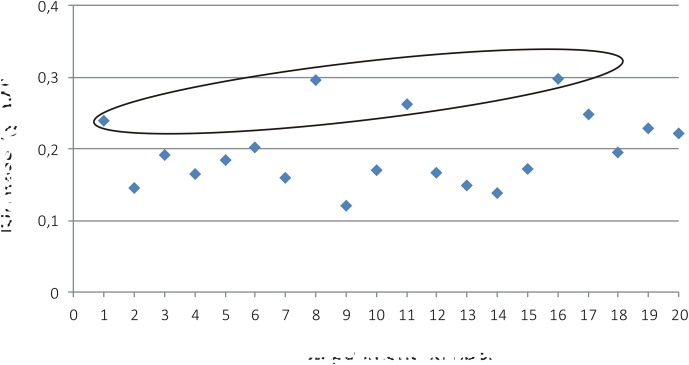
Biomass (g DW) from the experiments (**Table 1**) N1-N19 and N20 (original culture medium). Samples N17-N19 are the three replicate points. Four experiments resulted in even or higher biomass than with the original medium and are circled with blue (N1, N8, N11, and N16).

The BY-2 clone ‘Hulk’ was cultivated in media containing varying amounts of tested nutrients, as shown in **Table 1**. After 7 days cultivation, the samples were collected and biomass and accumulation of GFP-HFBI were measured. None of the tested factors was found to be significant either for accumulation of biomass or GFP-HFBI. When analyzed against GFP yield (g/l), a weak model was built with two significant factors CaCl_2_ and KH_2_PO_4_ (**Supplementary Figure S1**). However, model significance (*R*^2^) remained low with inadequate prediction power (*Q*^2^) (*R*^2^ = 0.60; *Q*^2^ = 0.42; model validity = 0.86, and reproducibility = 0.57, *p* < 0.05). For biomass, however, there were several experimental points which showed higher or similar values than the original culture medium (**Figure 4**).

The highest biomasses (N1, N8, N11, and N16; **Table 1**) achieved with the corresponding media (A1, A8, A11, and A16) were subjected to cost evaluation (**Table 2**). The most cost-efficient medium was A1 (**Table 2**), incurring only 37% of the cost of the original BY-2 medium. Next, the nutrients in A1 were further lowered in 10% intervals from 100 to 50% levels of the basic A1. Both BY-2 and ‘Hulk’ were assayed for biomass production and ‘Hulk’ for GFP accumulation.

**Table 2:** Estimated prices of selected media.

	Relative cost per liter (%)
Original medium	100
A1	37
A8	113
A11	80
A16	129


#### A1 Medium

**FIGURE 5:**
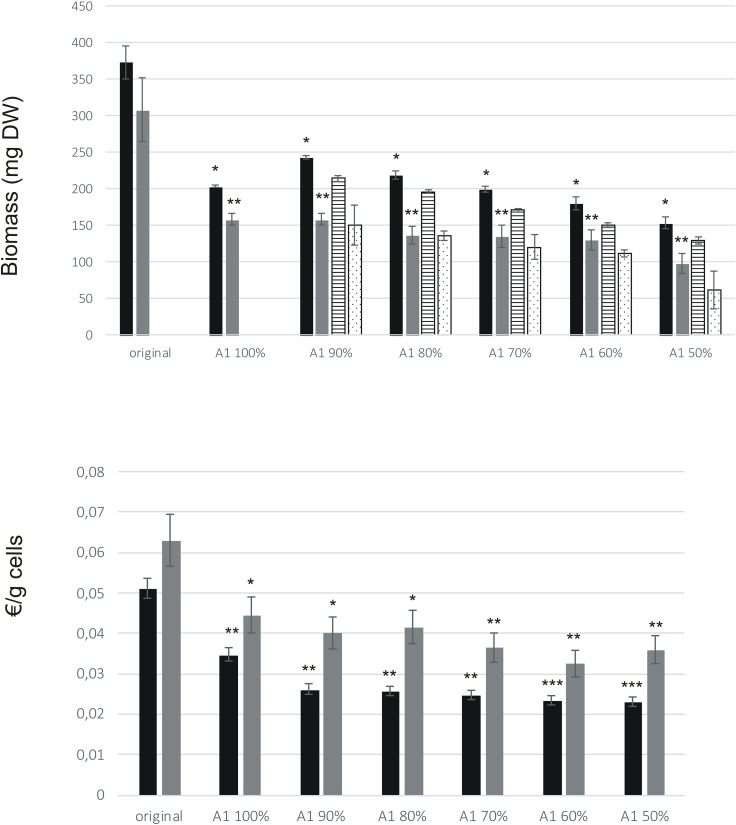
Growth of BY-2 and ‘Hulk’ in medium A1. Cultivation volume was 25 ml. Error bars represent SD of three biological replicates. Asterisks indicate the statistical difference against original medium, ^∗^*p* < 0.05, ^∗∗^*p* < 0.01, and ^∗∗∗^*p* < 0.001 (Student’s *t*-test, two-tailed). **(A)** Growth (mg DW) of BY-2 and ‘Hulk’ after 7 days of cultivation in medium A1 and its variations (A1 90% – A1 50%). Bar colors: black (BY-2), gray (‘Hulk’), striped (BY-2 in medium with fixed myo-inositol and 2,4-D), and dotted (‘Hulk’ in medium with fixed myo-inositol and 2,4-D). **(B)** Calculated cultivation medium costs for gram cells.

A1 medium contains only one-third of the macro salts and other studied factors compared to the levels in original medium. The biomass accumulation was significantly reduced in all A1 media for both BY-2 and ‘Hulk’ (*p* < 0.05, **Figure 5A**). However, when calculated against the cost, both BY-2 and ‘Hulk’ displayed economic gains in A1 medium and its variations (**Figure 5B**), and cost reduction in A1 media was significant for BY-2 (*p* < 0.01) and ‘Hulk’ (*p* < 0.05). The production costs due to cultivation medium per gram biomass for BY-2 and ‘Hulk’ in the A1 50% medium were 55 and 43% lower than with the original medium, respectively. In order to rule out the possible detrimental effects of altered hormonal and nutritional supplement levels, the growth was tested also by fixing the levels of 2,4-D and myo-inositol at the levels of the original culture medium in every test point (**Figure 5A**). However, fixed 2,4-D and myo-inositol levels did not benefit the growth. The observation was supported by the original DoE results where neither of the factors were significant for the growth.

**FIGURE 6:**
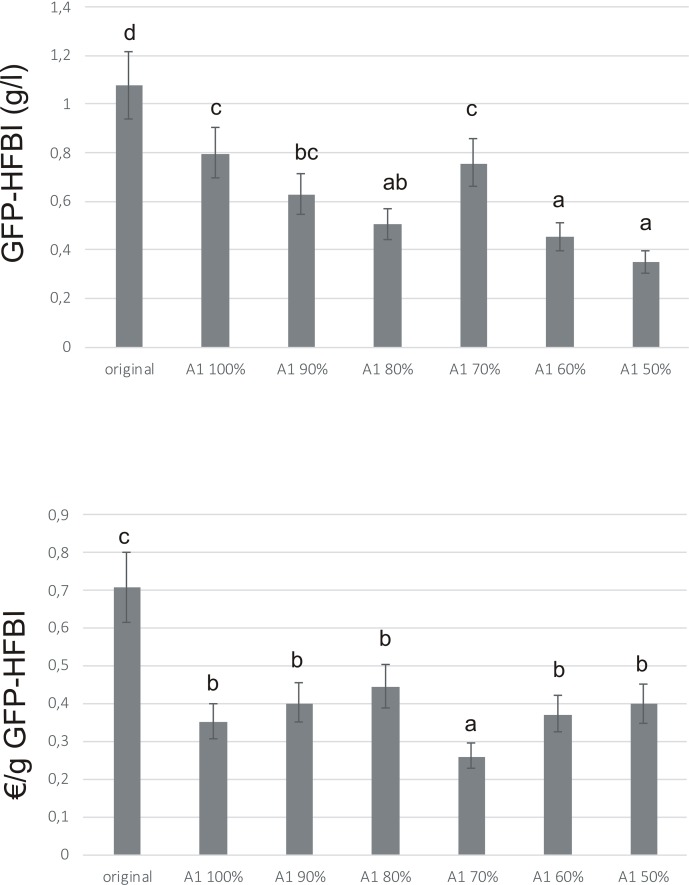
GFP-HFBI accumulation in ‘Hulk’ cultivated in medium A1. Error bars represent SDs of three biological replicates each measured three times (*N* = 9). Letters indicate statistical differences with *p* < 0.01. **(A)** GFP-HFBI yields (g/l) after 7 days of cultivation in medium A1 and its variations (A1 90% – A1 50%). **(B)** Calculated cultivation medium costs for producing gram GFP-HFBI.

GFP-HFBI yield was highest in original medium, due to best growth of ‘Hulk’ in that medium (**Figure 6A**). However, when calculated against the cost, A1 70% medium resulted in up to 69% cost reduction for production of gram GFP-HFBI, compared to the original medium (**Figure 6B**). A significant cost reduction was achieved with A1 in general, pointing out the importance of medium optimization in production process design.

## Discussion

The commercialization of plant-produced proteins has progressed slowly over the past 15 years since the first introduction of commercial product demonstrating feasibility (β-glucuronidase and avidin), and today there are more than 30 proteins that have been commercialized (Howard and Hood, 2014). Several factors determine whether a product is commercially viable, including technological as well as regulatory issues. One of the challenges in plant cell-based production systems has been high production costs resulting from relatively low production accumulation rates and expensive cultivation medium compared to other recombinant protein production platforms. In this work, the aim was to tackle the latter by optimizing the most costly culture medium nutrients without compromising the biomass and protein yields. Tobacco BY-2 cell culture was used as a production host for hydrofobin-fused GFP representing a typical platform for a model protein.

The GFP-HFBI titer of 1 g/l reached in this study with the BY-2 clone ‘Hulk’ is the highest titer of recombinant protein ever reported for BY-2 or any other plant suspension cell culture. The production level is comparable to the yields generally reported for yeasts and animal cells. The basic rule has been that a yield of 10 mg/l must be reached before the system can be considered for further R&D and the a yield of 100 mg/l should be in place to gain the desired profit margin so that plant-based recombinant protein expression system can be considered (Hellwig et al., 2004). Thus, this work clearly shows that BY-2 cells can be of economic interest for the production of recombinant proteins.

The factors causing the high expression level in ‘Hulk’ were not studied further and remains to be discovered. One reason behind the high accumulation rates can be that the integration of the GFP-HFBI encoding gene(s) is/are in optimal locus (loci), i.e., in a transcriptionally active spot of a chromosome. In addition, it might be that during the careful subcloning period of 3 years, some additional mutation or even somaclonal variations have gradually accumulated resulting in this superior production clone.

Cryopreservation of BY-2 ‘Hulk’ cell suspension culture was accomplished with a simple method earlier described by Ogawa et al. (2012) with slight modifications. As expected, cryopreservation did not alter the growth rate of the cell culture as cryopreservation by definition should arrest the cellular metabolism while it should allow the original properties of the cells to remain unaltered after cryostorage and thawing. Unexpectedly, the BY-2 cells expressing GFP-HFBI showed higher accumulation of recombinant protein after cryopreservation right after thawing when compared to original line. Since also the cells which were treated with cryoprotectants but not frozen showed increased yields, it is possible that in fact the stress caused by the treatment resulted in increased accumulation of GFP-HFBI. Actually, this could be one option to boost recombinant protein accumulation further in this system. However, the costs and feasibility in industrial scale operations should be carefully studied. The fact that the increase in protein accumulation was not permanent but stabilized to the levels of the non-treated cells along prolonged subculturing, supports the hypothesis of stress effect. Recently, we showed that organized cell cultures, such as hairy roots, can preserve their metabolic capacity even during very long continuous maintenance (Häkkinen et al., 2016). However, when cells are maintained in their undifferentiated state, genetic instability is often encountered in the form of rearrangements at chromosomal or gene level (Larkin and Scowcroft, 1981; Kaeppler et al., 2000). In this work, we showed that expression levels of GFP-HFBI remained stable in BY-2 cells for 3 months. The fluctuation in expression levels during longer period of time has earlier been monitored during 20 cultivation passages with the 10% standard error in GFP-HFBI/TSP ratio (**Figure 2E**), indicating good stability of the protein expression level in BY-2.

As observed by Holland et al. (2010), the nutrient requirements for high recombinant protein yields and high growth rate of the cells are contradictory, it was not possible to find conditions favorable to both responses without compromising either one. In accordance, the results from the current study show that since the growth in A1 medium remained lower than in the original medium, the total GFP-HFBI yield (g/l) remained as well lower. However, the aim of this study was to find the most economic culture medium for production of target protein and this goal was achieved with A1 medium resulting in a significant reduction of the culture medium cost, i.e., 69% reduction, per gram produced protein. In order to determine the real cost structure of the production of other target proteins, TEA (techno-economic assessment) or LCA (life cycle analysis) should be conducted with actual desired recombinant protein of commercial interest. However, this study demonstrates an example how significant cost savings can be achieved with reducing the consumption of most expensive components in the culture medium.

## Author Contributions

STH, AR, NN, and HR designed the experiments; NN performed the experiments for optimization and cryopreservation; AR and LR established the ‘Hulk’ line; JJ designed GFP-HFBI fusion system; STH, AR, and NN analyzed the data and STH and AR wrote the manuscript.

## Conflict of Interest Statement

The authors declare that the research was conducted in the absence of any commercial or financial relationships that could be construed as a potential conflict of interest.

## Supplementary Material

**FIGURE S1 |** Contour plot of the effects of KH_2_PO_4_ and CaCl_2_ on GFP yield (g/l) in ‘Hulk’. Model equation for GFP (g/l) = 0.0152 - 0.0001x + 0.0010y - 1.4464^∗^10^-6^y^2^, where x = [CaCl_2_]mg/l and y = [KH_2_PO_4_]mg/l. Model parameters: *R*^2^ = 0.60; *Q*^2^ = 0.42; model validity = 0.86 and reproducibility = 0.57, *p* < 0.05.

**TABLE S1 |** Amounts and list prices of the culture medium components. The components selected to the optimization study are highlighted in bold.aComponent concentration in the original medium. b List prices of Sigma for 1 kg of component (http://www.sigmaaldrich.com).
