# Supplementary figures and images for: Tobacco BY-2 Media Component Optimization for a Cost-Efficient Recombinant Protein Production
Source: Front Plant Sci. 2018 Jan 26;9:45. doi: 10.3389/fpls.2018.00045 (PMC5791008; doi:10.3389/fpls.2018.00045)

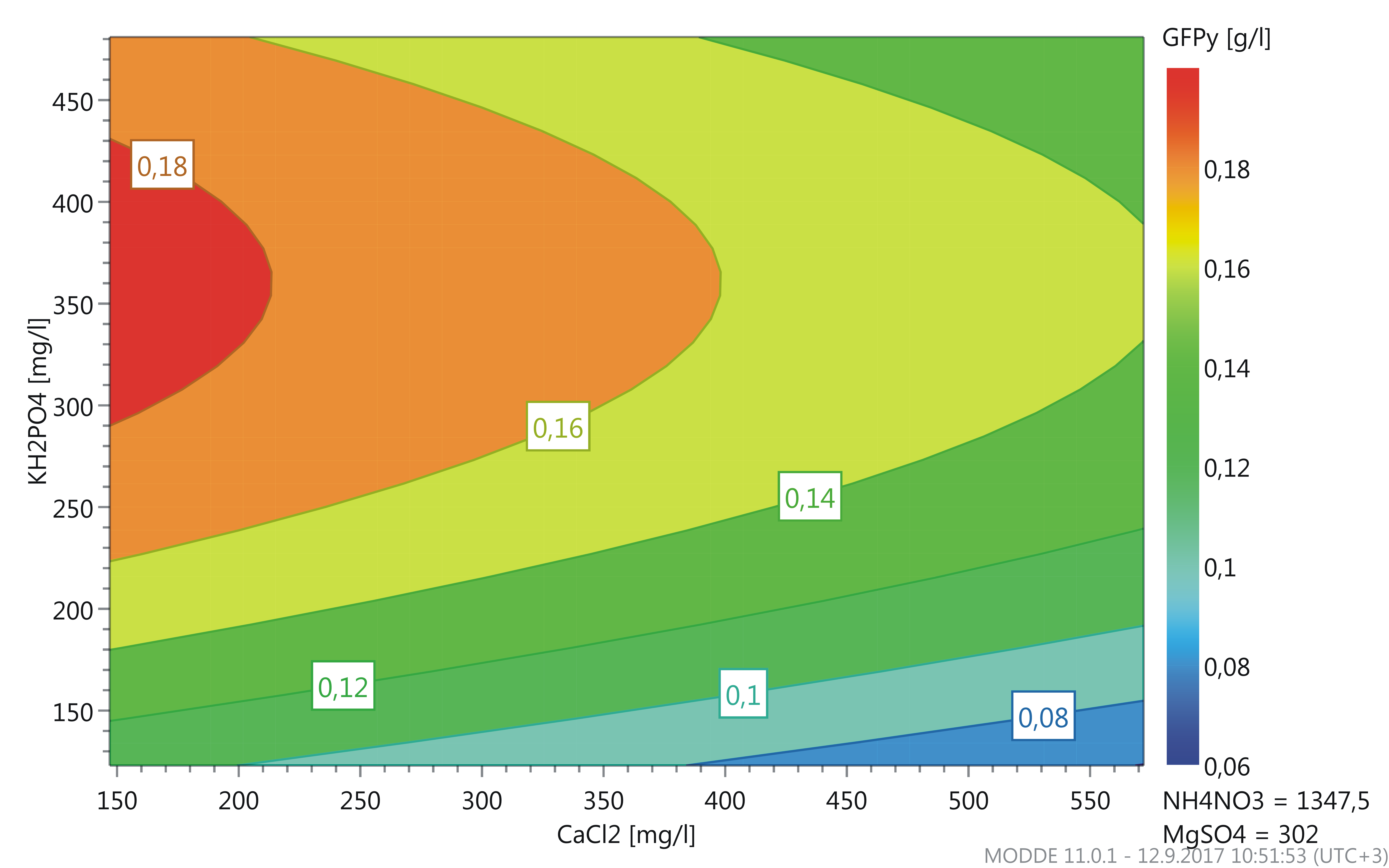

Supplement: Supplementary file 1 [file Image_1.TIF]
