# Supplementary material for: Tobacco BY-2 Media Component Optimization for a Cost-Efficient Recombinant Protein Production
Source: Front Plant Sci. 2018 Jan 26;9:45. doi: 10.3389/fpls.2018.00045 (PMC5791008; doi:10.3389/fpls.2018.00045)
Supplement: Supplementary file 2 [file Table_1.PDF]

Table S1. Amounts and list prices of the culture medium components. The components selected to the optimization study are highlighted in bold.

	mg/l [a]	€/kg [b]	€/l
**KH_2PO_4**	**370**	**122**	**0.045**
**NH_4NO_3**	**1650**	**143**	**0.236**
**KNO_3**	**1900**	**132**	**0.251**
**$MgSO_4$ $7H_2O$**	**370**	**185**	**0.068**
**$CaCl_2$ $2H_2O$**	**440**	**127**	**0.056**
$MnSO_4$ $4H_2O$	22.3	120	0.003
H_3BO_3	6.2	65	0.000
$ZnSO_4$ $7H_2O$	8.6	229	0.002
$NaMoO_4$ $2H_2O$	0.25	916	0.000
$CuSO_4$ $5H_2O$	0.025	280	0.000
$CoCl_2$ $6H_2O$	0.025	1088	0.000
KI	0.83	900	0.001
$FeSO_4$ EDTA	30	384	0.014
sucrose	30000	0.7	0.021
**myo-inositol**	**100**	**624**	**0.062**
thiamine-HCl	1	1325	0.001
**2,4-D**	**0.2**	**14320**	**0.003**

[a] Component concentration in the original medium

[b] List prices of Sigma for 1 kg of component (www.sigmaaldrich.com)
